# Association between Etomidate Use for Rapid Sequence Intubation and Adrenal Insufficiency in Sepsis

**DOI:** 10.7759/cureus.13445

**Published:** 2021-02-19

**Authors:** Joaquin A Cagliani, Andres Ruhemann, Ernesto Molmenti, Candace Smith, Gene Coppa, Rafael Barrera

**Affiliations:** 1 Anesthesiology, State University of New York (SUNY) Downstate Medical Center, New York, USA; 2 Internal Medicine, Instituto Alfredo Lanari, Buenos Aires, ARG; 3 General Surgery, Northwell Health, New York, USA; 4 Internal Medicine, College of Pharmacy and Health Sciences - St. John's University, New York, USA

**Keywords:** etomidate, rapid sequence intubation, critical care anesthesiology, primary adrenal insufficiency

## Abstract

Background

The risk of adrenal insufficiency (AI) in using single-dose etomidate for intubation among patients with sepsis remains controversial. Our aim was to assess the prevalence of AI and characterize the risk factors in patients who received etomidate for rapid sequence intubation (RSI).

Methods

This is a retrospective study of prospectively-acquired data evaluating surgical intensive care unit (SICU) patients who developed respiratory failure undergoing RSI.

Results

Of the 44 adult SICU patients who developed respiratory failure, 34 patients received etomidate. The average age for the total cohort, for the patients that received etomidate and for those who did not, was 70.91 ± 14.92, 72.82 ± 13.61 years and 64.40 ± 15.93, respectively. Twenty-four patients of the total cohort (54.55%) developed AI; 26 had septic shock (59.09%), and 16 patients had AI and septic shock (36.36%). There was no statistical significance between the incidence of AI in patients who received etomidate (47%) and those who did not (80%). However, in the subset of patients who received etomidate for RSI, there was a non-significant trend toward increased incidence of AI in those who were septic compared to those who were not (p = 0.06).

Conclusion

A single dose of etomidate used for RSI in SICU patients is not associated with the development of AI or mortality. However, a trend was shown, although not statistically significant, towards the development of AI in septic patients. High-quality and adequately powered randomized control trials (RCTs) are warranted.

## Introduction

Rapid sequence intubation (RSI) is being increasingly used in the operating room, emergency room, and intensive care units as the primary approach to ensure proper airway placement [1]. RSI involves the use of a sedative and a neuromuscular blocking agent in quick succession. According to the National Emergency Airway Registry (NEAR) study, etomidate is the most frequently used induction agent for RSI in adults [2]. Because of etomidate’s rapid onset, short half-life, and favorable risk-benefit profile, it has become one of the primary induction agents for emergency airway management and is also being used for induction of general anesthesia [3].

The recommended dose of etomidate for RSI is 0.2 to 0.4 mg/kg over a period of 30-60 seconds. It is an ultra-short acting non-barbiturate hypnotic, that induces loss of consciousness within 5-15 seconds. Etomidate is a carboxylate imidazole that produces rapid induction of anesthesia with minimal cardiovascular effects. The peak effect occurs at one minute and lasts for 5-15 minutes. The half-life is 75 minutes and is metabolized primarily by the liver and plasma esterase, with 75% excreted in the urine, 13% in feces, and 10% in bile [4]. Although etomidate has a favorable safety profile [5,6], it is also known to inhibit 11-ß-hydroxylase and 17-α-hydroxylase [7], enzymes necessary for steroid production. By inhibiting the conversion of cholesterol to cortisol by a reversible and concentration-dependent blockade, etomidate leads to decreased plasma cortisol and aldosterone levels. The resulting adrenal suppression can occur approximately 30 minutes after induction, and lasts 5 to 15 hours, but is reported to be reversible and usually resolves in less than 12 hours [4].

Over the past few years, several retrospectives studies have documented the development of adrenal insufficiency (AI) in critically injured patients who received etomidate for RSI [5]. According to the published literature, a single bolus of etomidate was associated with AI in 80% of critically ill patients at 12 hours. However, it decreased to 9% at 48 hours and to 7% at 72 hours [8]. While several studies have implicated etomidate in causing increased mortality and AI in critically ill patients [9-12], other studies found that etomidate use in patients with severe sepsis and septic shock was correlated with decreased mortality [13,14].

In light of the lack of consensus and conflicting data, we conducted a retrospective study to evaluate whether the use of etomidate for RSI is appropriate in the critically ill patient population, specifically in patients with sepsis. We examined the usage patterns, risk of developing AI, and in-hospital mortality rate associated with etomidate in this population.

## Materials and methods

Study design and participants

This is a retrospective study of prospectively acquired data evaluating patients who developed respiratory failure and received etomidate for RSI. The study was conducted at a 13-bed surgical intensive care unit (SICU) in an academic medical center.

All patients in this study were aged 18 years or older, admitted to the SICU and cared for by a dedicated surgical critical care team. Patients who had a history of AI, adrenal injury or had been on long-term corticosteroids were excluded from the study.

We documented whether patients received etomidate during endotracheal intubation for respiratory distress. The decision to use etomidate was based on the clinical expertise of the admitting and/or surgery physician after discussion with the SICU physician. These patients were then further queried for development of AI and/or septic shock.

Clinical criteria for AI included random cortisol levels less than 25 mcg/dl and/or with concurrent hypotension with mean arterial blood pressure (MAP) <65 mm Hg. Septic shock was defined by persistent arterial hypotension despite adequate fluid resuscitation in septic patients with concurrent requirement of vasopressor agents to maintain MAP > 65 mm Hg. This study was approved by the Northwell Health System Institutional Review Board.

Data collection

Data were extracted from the medical record of the patients meeting inclusion criteria. Baseline demographic variables were collected, which included age, sex, body mass index (BMI), race and medical comorbidities including diabetes mellitus, hypertension, end-stage renal disease, coronary artery disease, cerebrovascular accident or malignancy. Etomidate dosing and date, and time of administration as well as potential baseline risk factors for the development of AI such as adrenal injury, coagulopathy, hemorrhagic shock, septic shock, traumatic brain injury, and vasopressor dependence were also documented. Concomitant RSI medications such as succinylcholine, midazolam, rocuronium and steroids administration with hydrocortisone or dexamethasone was also collected.

Outcomes

We evaluated the development of AI and/or septic shock in the adult SICU patients and stratified them by etomidate exposure. Secondary outcomes recorded were concurrent presence of traumatic brain injury, coagulopathy, length of hospital stay, length of SICU stay, use of other induction medications used during RSI, and in-hospital mortality.

Statistical analysis

Data are expressed as mean (SD) for normally distributed variables, median (interquartile range) for non-Gaussian quantitative variables, and as numbers and percentages for categorical variables. Normality of the distribution was checked using the Kolmogorov-Smirnov test. The association between AI and etomidate treatment as well as the presence of septic shock were analyzed using chi-square or Fisher exact tests when appropriate. The Cochran-Mantel-Haenszel test was applied to explore the conditional independence between AI and septic shock after controlling for etomidate treatment status. A 2-tailed p value <0.05 was considered statistically significant. Statistical analysis was performed using the SAS system, version 9.2 (SAS Institute, Cary, North Carolina).

## Results

Over the four-year study period, 53 patients underwent RSI in the SICU. However, only 44 patients met inclusion criteria. Nine patients were excluded due to missing data (n=4), history of AI (n=2) and long-term history of corticosteroids (n=3). Of the 44 patients, 77.3% (n=34) received a single dose of etomidate for RSI, either alone or in combination with other induction agent including: rocuronium (n=17), succinylcholine (n=12), propofol (n=8), lorazepam (n=1), and/or midazolam (n=2), fentanyl (n=1), cisatracurium (n=2). Of the 10 patients who did not receive etomidate. eight received midazolam, six received propofol, nine received fentanyl, seven received rocuronium, two received succinylcholine, one received pancuronium, one received sufentanil. The mean age (SD) of the entire cohort was 70.91 (± 14.42) years and 56.8% were male. Baseline characteristics were similar between study groups. There was no difference in age, gender, or race between patients who received etomidate and those who did not. Diabetes mellitus was more commonly seen in the non-etomidate group as compared to the etomidate group (60% vs 20.5%, respectively p=0.02) (Table 1). Of all patients included in our study, 26 (59.1%) were septic at the time of intubation, with no difference in distribution between etomidate (64.7%) and non-etomidate groups (40%), as shown in Table 1.

**Table 1:** Baseline Characteristics BMI: body mass index; SD: standard deviation; DM: diabetes mellitus; HTN: hypertension; CKD: chronic kidney disease; CAD: coronary artery disease; CVA: cerebral vascular accident. *Statistical significant

Characteristics		Etomidate Group (n=34)		No Etomidate Group (n=10)		P value	
Age (y), Mean ± SD		72.82 ± 13.61		64.40 ± 15.93		0.20	
Sex Male, n (%)		19 (55.8)		6 (60)		1.00	
BMI (kg/m^2^), Mean ± SD		30.12 ± 8.22		30.69 ± 4.46		0.30	
Race, n (%)	African American		9 (26.4)		4 (40)	0.64	
	Asian		6 (17.6)		1 (10)		
	White		18 (52.9)		5 (50)		
	Other		1 (2.9)		0 (0)		
Comorbid Conditions, n (%)	DM		7 (20.5)		6 (60)	0.02*	
	HTN		26 (76.5)		9 (90)	0.66	
	CKD		11 (32.3)		3 (30)	1.0	
	CAD		12 (35.3)		4 (40)	1.0	
	CVA		4 (11.8)		0 (0)	0.56	
	Malignancy		10 (31.3)		4 (40)	0.7	
Septic Shock, n (%)		22 (64.7)		4 (40)		0.14	

AI developed in 23 out of 44 patients (52.3%), with 16 (47.1%) in the etomidate group and eight (80%) in the non-etomidate group. The difference was not statistic significant (p=0.08) (Table 2). Etomidate administration was not associated with in-hospital mortality in our sample of SICU patients (38.2% in etomidate group vs 40.0% in non-etomidate group, p=1.0). SICU and hospital length of stay (LOS) was not statistically different among the etomidate vs the non-etomidate groups (SICU LOS 9.5 days’ vs 3.0 days, respectively. p=0.13), (hospital LOS 19.5 vs 7.0, respectively. p=0.14). 

**Table 2:** Clinical Outcomes IQR: interquartile range; SICU: surgical intensive care unit; LOS: length of stay.

Outcome	Etomidate Group (n=34)	Non Etomidate Group (n=10)	P value
Adrenal Insufficiency, n (%)	16 (47.1)	8 (80)	0.08
Mortality, n (%)	13 (38.2)	4 (40)	1.0
SICU LOS (d), Median (IQR)	9.5 (6.5 - 16.5)	3.0 (1 - 27.7)	0.13
Hospital LOS (d), Median (IQR)	19.5 (12.75 - 31.25)	7.0 (3.0 - 47.0)	0.14

Sepsis has been suggested as a risk factor for developing AI in patients receiving etomidate for RSI. A subgroup analysis of patients with sepsis who received etomidate (n=22, 64.7%) found a trend toward increased RAI among septic patients (59.1% vs 16.7%, P=0.06), as shown in Table 3. However, administration of etomidate was not associated with increased in-hospital mortality, SICU LOS, or hospital LOS. 

**Table 3:** Subgroup Analysis of Patients Who Received Etomidate IQR: interquartile range; SICU: surgical intensive care unit; LOS: length of stay.

Outcome	Septic (n=22)	Non septic (n=12)	P value
Adrenal Insufficiency, n (%)	13 (59.1)	2 (16.7)	0.06
Mortality, n (%)	8 (36.4)	2 (16.7)	0.44
SICU LOS (d), Median (IQR)	9.5 (4.5 - 14.5)	9.5 (2.5 - 16.5)	0.37
Hospital LOS (d), Median (IQR)	20.0 (7.0 - 33.0)	22.0 (11.0 - 33.0)	0.81

There were no associations discovered between BMI, malignancy, the number of comorbidity conditions, and the development of AI in septic and non-septic patients in the SICU.

## Discussion

It is now widely accepted that the use of etomidate is associated with increased risk of AI. However, controversy remains whether it is associated within the subset of septic patients [15]. While previous studies have shown that etomidate is not associated with higher rates of AI in septic patients [16,17], several studies [18,19], including a recent meta-analysis [20] with a total of 865 subjects from five different studies reported an increased likelihood of developing AI in septic patients. In congruency, we found a trend, although not statistically significant, towards the development of AI in the overall cohort as well as in the patients with baseline sepsis with etomidate use for RSI.

In 2014, Freund et al. published an ancillary study to the KETASED (ketamine versus etomidate during rapid sequence intubation: consequences on hospital morbidity) study, demonstrating that etomidate was associated with AI in patients with critical illness of various etiologies including coma, shock, and acute respiratory failure. The KETASED study was conducted in an out-of-setting hospital, where the majority of intubations were performed in comatose patients and very few in trauma or septic patients. Although they also examine the risk of RAI in the subgroup of patients with trauma or sepsis, their study was not powered to detect differences in AI and mortality in this subgroup of patients [6].

Similar to a recent systemic review and meta-analysis published in 2015 by Gu et al. [10], we did not find any statistically significant difference in the in-hospital mortality associated with etomidate use in the overall cohort or the sepsis cohort.

The safety of etomidate use for RSI will continue to be controversial unless we have a high-powered randomized control trial (RCT) that analyzes subgroups of patients with clearly defined risk factors. Until we have conducted these high-quality studies that generate accurate results, we should analyze each patient on an individual basis, understand the risk factors for morbidity and mortality, make judgments based on evidence, and then decide our next course of treatment.

It is important to note that all studies analyzing AI in critically ill patients have one limitation in common: critically ill patients are in a catabolic state. Free cortisol is bound to cortisol binding protein (CBP), and in prolonged catabolic states protein synthesis is reduced and critically ill patients, septic or not, will have a decrease in CBP and therefore, a decrease in total cortisol (cortisol bound to CBP plus free cortisol). AI is defined as a low cortisol response despite adrenocorticotropic hormone (ACTH) administration. RCT and meta-analysis fail to take into account confounding variables such as variable cortisol baselines, and concentrations of albumin and corticosteroid-binding globulin (CBG), which tend to fall in critical illness, without actually reducing free cortisol levels. In addition, our data may be underpowered and lack long-term follow up. Therefore, the scientific and medical community needs high-powered prospective RCTs to deliver the most accurate and complete results to assess RAI, mortality, and long-term outcomes.

## Conclusions

In summary, the present study suggests that a single dose of etomidate used for RSI in SICU patients is not associated with development of AI or mortality. However, a trend appeared, although not statistically significant, towards development of AI in septic patients. The use of etomidate in septic patients should be used with cautions. High-quality and adequately powered RCTs are warranted.
